# Immune Checkpoint Inhibitor-induced Fanconi Syndrome

**DOI:** 10.7759/cureus.7686

**Published:** 2020-04-16

**Authors:** Saira Farid, Hira Latif, Chanigan Nilubol, Chul Kim

**Affiliations:** 1 Internal Medicine, Medstar Washington Hospital Center-Georgetown University Hospital, Washington, D.C, USA; 2 Hematology/Oncology, Medstar Washington Hospital Center, Washington, D.C, USA; 3 Nephrology, Georgetown University Hospital, Washington, D.C, USA; 4 Oncology, Georgetown University Hospital, Washington, D.C, USA

**Keywords:** checkpoint inhibitor therapy, fanconi syndrome, nivolumab, ipilimumab

## Abstract

Immune checkpoint inhibitors (ICI) have been approved by the Food and Drug Administration (FDA) for use in many solid tumors and hematological malignancies. Immune-related adverse events (irAEs) are potential side effects that can arise during or after treatment with ICI therapy. We describe a case of ICI-induced Fanconi syndrome in a 58-year-old man with extensive-stage small-cell lung cancer (ES-SCLC), who had disease progression after initial chemotherapy and radiation. He was started on nivolumab and ipilimumab as second-line treatment. Three weeks into the therapy, he developed abdominal pain with grade 3 transaminitis and required steroids and mycophenolate for presumed autoimmune hepatitis. Subsequently, he presented with worsening abdominal pain and was found to have an enlarging right adrenal mass. Laboratory work-up revealed a white blood cell (WBC) count of 17 K/µL, aspartate aminotransferase (AST)/alanine aminotransferase (ALT) 99/210 U/L, direct bilirubin 2.8 mg/dL, blood urea nitrogen (BUN) 43 mg/dL, Cr 2.31 mg/dL (baseline: 1.1 mg/dL), phosphorus 2.3 mg/dL, and glucose 303 mg/dL with metabolic acidosis. There was no evidence of urinary tract obstruction. Urinary findings were notable for glucosuria (>500 mg/dL), fractional excretion of phosphorus and uric acid of 56% (normal range 10%-20%) and 75% (normal range 7%-10%), respectively. He was started on intravenous (IV) bicarbonate and methylprednisolone. Fanconi syndrome with proximal tubular damage secondary to ICI therapy was diagnosed. He was discharged on oral bicarbonate and steroid taper. On follow-up after four weeks, his renal function recovered to baseline.

## Introduction

Immune checkpoint inhibitors (ICIs) blocking cytotoxic T-lymphocyte antigen 4 (CTLA-4) and the programmed cell death protein 1/ programmed cell death ligand 1 (PD-1/PD-L1) axis have been approved by the U.S. Food and Drug Administration (FDA) for use in several solid and hematological malignancies [1]. With the widespread use of these agents, immune-related adverse events (irAEs) have been increasingly encountered in clinical practice. Reported renal adverse events (AEs) described so far include acute interstitial nephritis, minimal change disease, and immune complex glomerulonephritis [2-3]. In this report, we present a case of nivolumab/ipilimumab-induced Fanconi syndrome, which was treated with steroids and sodium bicarbonate. To our knowledge, our report is the first to describe nivolumab/ipilimumab-induced renal AEs manifesting as Fanconi syndrome. This article was first presented as an abstract at the ICAHO meeting, 2019. (Farid. S, Latif. H, Kim, C; Immune Checkpoint Inhibitor-induced Fanconi Syndrome; International Conference on Advances in Hematology and Oncology; June 28, 2019)

## Case presentation

A middle-aged male with a history of tobacco use was diagnosed with extensive-stage small-cell lung cancer (ES-SCLC) after a biopsy of a left mediastinal mass, with right adrenal involvement. He completed six cycles of cisplatin and etoposide, followed by thoracic and prophylactic cranial radiation. A follow-up computed tomography (CT) scan after three months showed an interval progression of the disease in the left lung and the right adrenal gland. He underwent a positron emission tomography-computed tomography (PET-CT) scan, which revealed several new metastases to lymph nodes in the neck, chest, abdomen and pelvis, bones and pancreas. Brain MRI showed a small enhancing lesion in the left cerebellum. He was started on nivolumab (3 mg/kg) and ipilimumab (1 mg/kg) followed by CyberKnife (Accuray Incorporated, Sunnyvale, California) treatment for the brain lesion. Three weeks into the treatment, he developed abdominal pain with grade 3 transaminitis, which was thought to be secondary to ICI toxicity. He was treated with intravenous methylprednisolone (1 mg/kg/twice a day) for possible immune-related hepatitis without improvement in transaminitis. Nivolumab/ipilimumab was subsequently stopped and mycophenolate (1 g/twice a day) was added on top of oral prednisone taper (70 mg/twice a day).

Ten days after discharge, he presented to the emergency department with right upper quadrant pain, fevers, and tachycardia. Laboratory findings are illustrated in Table 1. Abdominal ultrasound revealed intrahepatic and extrahepatic ductal dilatation. With worsening bilirubin of up to 5.5 mg/dL, he was started on vancomycin and piperacillin/tazobactam for potential cholangitis. For transaminitis, he was re-started on intravenous methylprednisolone (1 mg/kg/twice a day). MRI abdomen/pelvis and magnetic resonance cholangiopancreatography (MRCP) revealed severe biliary dilatation due to common bile duct stricture related to the mass effect from adrenal metastasis as well as pancreatic/peripancreatic nodal disease. Endoscopic retrograde cholangiopancreatography (ERCP) was performed with stent placement, which resolved his bilirubinemia.

**Table 1:** Laboratory findings at baseline, at presentation, and at a four-week interval AST: Aspartate Aminotransferase (AST); ALT: Alanine Aminotransferase; BUN: Blood Urea Nitrogen; pCO2: Partial Pressure of Carbon Dioxide; FENa: Fractional Excretion of Sodium; FePhos: Fractional Excretion of Phosphorus; FEUrate: Fractional Excretion of Urate

Labs/normal range	Baseline	At presentation	At 4 weeks
Serum			
White blood cells (k/µL)/(4-10.8)	5.2	17	4.7
AST (U/L)/(3-34)	30	99	30
ALT (U/L)/(15-41)	21	210	28
BUN (mg/dL)/(9-20)	28	43	32
Cr (mg/dL)/(0.66-1.50)	1.0	2.3	1.0
Sodium (mmol/L)/(137-145)	141	141	139
Potassium (mmol/L)/(3.5-5.1)	4.1	4.5	4.5
Chloride (mmol/L)/(98-107)	104	112	107
Bicarbonate (mmol/L)/(21-32)	24	12	22
Phosphorus (mg/dL)/(2.5-4.5)	3.6	2.3	2.6
Glucose (mg/dL)/(65-140)	126	303	187
Anion gap/(5-15)	8	6	10
Arterial			
pH/(7.32-7.42)		7.35	7.40
pCO2 (mmHg)/40		23	39
Urine			
Glucose (mg/dL)	Normal	>500	Normal
FENa (%)		2	1.9
FEPhos (%)/(10-20)		56	21
FEUrate (%)/(7-10)		75	20

The metabolic acidosis and hypophosphatemia along with glucosuria, phosphaturia, and high urate excretion led to a diagnosis of Fanconi syndrome (FS) representing proximal tubular damage. There was no other identifiable medication, which may have contributed to this degree of renal and hepatic injury. A renal biopsy was not performed, as the patient’s kidney function improved with corticosteroids. Renal ultrasonography did not show any evidence of urinary tract obstruction. Intravenous bicarbonate for metabolic acidosis was initiated. He was continued on supportive care and discharged on oral bicarbonate and steroid taper. His renal function returned to baseline (serum Cr of 1.0 mg/dL) at a follow-up visit four weeks later. The patient subsequently received a third-line regimen, topotecan but, unfortunately, his disease progressed after two cycles, and he transitioned to comfort care.

## Discussion

Cytotoxic T-lymphocyte antigen 4 (CTLA-4) and programmed cell death protein 1 (PD-1) are immune checkpoints that are expressed by T lymphocytes. CTLA-4 prevents T-cell activation by competing with CD28 for its ligand, B7, and PD-1 downregulates effector T-cell function by engaging its two ligands, PD-L1 and PD-L2 [4]. ICIs are monoclonal antibodies that enhance the immune response against tumors by targeting these receptors. ICIs have been approved as therapeutic agents for many solid and hematologic malignancies, including small cell lung cancer, non-small lung cancer, metastatic melanoma, renal cell carcinoma, and Hodgkin lymphoma [1,5-6]. Distinct immunological effects occurring during a combined blockade have proven to be effective in advanced melanoma and renal cell carcinoma, resulting in improved outcomes that lead to the approval of combination immunotherapy [7-8].

The toxic effects associated with ICIs may affect any organ. Checkpoint inhibitory pathways are protective in normal physiology and work to suppress the immune response against self-antigens. When these pathways are blocked by ICIs, the normal self-protective response is lost, and enhanced inflammation can cause immune invasion and damage of normal tissues. While not completely understood, this is the likely cause of the irAEs seen with ICI therapy [9]. Most frequently, these immune-related adverse events (irAEs) affect the skin, liver, colon, thyroid, and lungs and may affect other organs as well [10-11].

Cortazar et al. analyzed data from published phase 2 and 3 clinical trials of patients with adverse renal outcomes on ICI therapy and found the overall incidence of acute kidney injury (AKI) to be 2.2% in 3695 patients. The incidence of grade 3 or 4 AEs or the need for dialysis was 0.6% [12]. A phase 3 trial (CheckMate 067) conducted to evaluate the safety and efficacy of monotherapy versus a combination of nivolumab and ipilimumab in patients with metastatic melanoma, renal AEs were more common in patients who received combination therapy with ipilimumab and nivolumab (4.9%) than in patients who received monotherapy with ipilimumab (2.0%) or nivolumab (1.9%) [12]. It has also been reported that combination therapy triggers an earlier and higher grade of adverse events than monotherapy [10].

The most commonly reported renal AEs associated with ICI therapy is interstitial nephritis [13]. There are also case reports of lupus nephritis, granulomatous nephritis, and minimal change disease [3,14-16]. Low-grade kidney injury has been reported in 25%-29% of patients taking certain ICIs [17]. Renal toxicity from ICIs is usually asymptomatic. Rising creatinine (100%) and pyuria (68%) may be the only clinical clue while other findings, such as hematuria (16%), eosinophilia (21%), worsening hypertension (11%), peripheral edema and anorexia, have been reported [12,18]. The onset of renal injury seen with PD-1 inhibitors is usually three to 10 months and with anti-CTLA-4 inhibitors, it is around two to three months [12]. These reported time frames are from studies that report ICI-induced acute interstitial nephritis (AIN). The mechanism of ICI-induced AIN is thought to be secondary to the loss of tolerance against endogenous kidney antigens, as opposed to a delayed-type hypersensitivity reaction with other drugs. Our patient presented with FS within four weeks of starting the ICIs. The mechanism for FS secondary to ICI has not been well-described. However, there have been several publications describing the potential mechanisms of tubulointerstitial disease and FS in autoimmune diseases. Belmouaz et al. described a case of tubulointerstitial nephritis and FS in a patient with Behcet disease. In their case, immunopathological and electron microscopic studies showed predominant T-cell infiltration of the renal interstitium with severe proximal tubulitis, leading to a hypothesis that lymphocytic infiltration of the tubular epithelium is likely to be involved in the inhibition of proximal tubular function in tubulointerstitial nephritis-associated autoimmune disorders [19]. T-cell receptor analyses and immune profile characterization may enable us to risk-stratify patients and better manage these complications in the future.

The efficacy of glucocorticoids in the treatment of AIN remains controversial due to conflicting observational studies and the absence of a randomized trial [11,20]. Cortazar et al. reported 90% of patients treated with glucocorticoids had complete or partial recovery of renal function, as compared to patients who were managed conservatively. Due to limited data, the optimal dosing regimens and treatment duration remain unknown. This study also demonstrated that patients who did not respond to steroid treatment had moderate to severe interstitial fibrosis on renal biopsy. Hence, for complete recovery from ICI-induced AIN, as with other causes, early recognition of injury and prompt treatment may be necessary. Our patient was managed by a steroid taper and his kidney function recovered.

## Conclusions

In conclusion, the high prevalence of ICI use in multiple cancers warrants attention to the adverse effects secondary to these ICIs, including renal complications. The case in this report adds to a growing body of literature suggesting the need for close monitoring of renal toxicities during an immune checkpoint blockade. Monitoring of kidney function during ICI therapy is vital for the early detection and management of renal AEs and the prevention of severe, irreversible renal damage. The early initiation of immunosuppressive therapy and other supportive measures may result in favorable outcomes.
